# Use of clinical and computed tomography findings to assess long-term unsatisfactory outcome after femoral head and neck ostectomy in four large breed dogs

**DOI:** 10.1186/s13028-018-0382-8

**Published:** 2018-05-10

**Authors:** Ciprian Ober, Cosmin Pestean, Lucia Bel, Marian Taulescu, Joshua Milgram, Adrian Todor, Rodica Ungur, Mirela Leșu, Liviu Oana

**Affiliations:** 1 Department of Surgical Techniques, University of Agricultural Sciences and Veterinary Medicine, 3-5 Mănăştur Street, 400372 Cluj-Napoca, Romania; 2 Department of Pathology, University of Agricultural Sciences and Veterinary Medicine, 3-5 Mănăştur Street, 400372 Cluj-Napoca, Romania; 3 Department of Surgery, Koret School of Veterinary Medicine, P.O. Box 12, 76100 Rehovot, Israel; 4 Department of Orthopedics and Traumatology, University of Medicine and Pharmacy Iuliu Hatieganu, 8 Babeş Street, 400012 Cluj-Napoca, Romania; 5 Department of Balneo-physio-kinetotherapy and Recuperation, University of Medicine and Pharmacy Iuliu Hatieganu, 8 Babeş Street, 400012 Cluj-Napoca, Romania; 6 University of Agricultural Sciences and Veterinary Medicine, 3-5 Mănăştur Street, 400372 Cluj-Napoca, Romania

**Keywords:** Computed tomography, Dog, Femoral head and neck ostectomy, Hip joint

## Abstract

Femoral head and neck ostectomy (FHNO) is a salvage surgical procedure intended to eliminate hip joint laxity associated pain in the immature dog, or pain due to secondary osteoarthritis in the mature dog. The outcome of the procedure is associated with the size of the dog but the cause of a generally poorer outcome in larger breeds has not been determined. The objective of this study was to assess the long-term results of FHNO associated with unsatisfactory functional outcome by means of clinical examination and computed tomography (CT) scanning. Four large mixed breed dogs underwent FHNO in different veterinary clinics. Clinical and CT scanning evaluations were carried out long time after the procedures had been done. Hip pain, muscle atrophy, decreased range of motion and chronic lameness were observed at clinical examination. Extensive remodelling, unacceptable bone-on-bone contact with bony proliferation involving the femoral neck and acetabulum, but also excessive removal with bone lysis were observed by CT scanning. Revision osteotomy was performed in one dog. Deep gluteal muscle interposition was used, but no improvements were observed postoperatively. This is the first report on the evaluation of three-dimensional CT reconstructions of the late bone remodelling associated with poor clinical outcome in large dogs. The study shows that FHNO could lead to severe functional deficits in large breed dogs. An extensive follow-study is necessary to more accurately determine the frequency of such complications.

## Findings

Femoral head and neck ostectomy (FHNO) is a salvage surgical procedure for hip dysplasia intended to eliminate hip joint laxity associated pain in the immature dog, or pain due to secondary osteoarthritis in the mature dog [1]. The procedure is relatively straightforward and has been the topic of several studies [2–8]. The perception that function after FHNO is better in small dogs and cats compared with larger dogs is based upon a widely accepted presumption, i.e. that the ability to compensate for the mechanical disadvantages of an absent coxofemoral articulation dependents on body weight, with lighter animals having an advantage [3, 4, 6, 9], but functional disabilities have also been reported in many small breed dogs and cats subjected to FHNO [2, 10]. Some studies suggest that bone-on-bone contact from inadequate excision or postoperative formation of enthesophytes or ectopic bone is the primary cause of poor outcome [9, 11]. Other authors suggest that the bone contact is not sufficient to explain differences in clinical outcome [5]. Muscle transpositions using the biceps femoris muscle [7, 9, 12], the deep gluteal muscles [2] and a vascularized rectus femoris muscle sling have been described, but are no longer recommended because of potential ischiatic nerve damage [12, 13]. Kinetic gait analysis has failed to demonstrate improvement in weight bearing when interpositions have been used [14, 15]. The aim of this study is to report post-ostectomy clinical and CT findings associated with functional disabilities after FHNO in four large breed dogs.

Four mixed breed dogs were presented to the Department of Surgical Techniques, Faculty of Veterinary Medicine, Cluj-Napoca, Romania for revision of a failed FHNO. According to the owners, the reasons for FHNO were chronic coxofemoral luxation (2 dogs), fracture of the femoral neck (1 dog) and osteoarthritis due to hip dysplasia (1 dog). The dogs had an average age of 5 years (range 2–9 years) at the time of FHNO. Mean body weight was 30.7 kg (range 27–31 kg). Examinations of the patients were carried out 12–15 months after surgery. Lameness, pain and muscle atrophy scores were assessed after physical examination (Table 1). Crepitus was observed in two patients (cases 2 and 3).

**Table 1:** Clinical data, lameness, pain and musculature scores (veterinary locomotor and physical examination questionnaire)

Case	Case data	Pain score	Lameness score	Musculature score	Reason to perform FHNO
1.	Four years old, 31 kg, male	Discomfort during extension of the hip (turns itself) (score 1)	Persistent severe weight-bearing lameness (score 4)	Decreased musculature of the hip and thigh regions (score 1)	Chronic coxofemoral luxation
2.	Five years old, 27 kg, female	Severe pain during extension of the hip (attempts to bite) (score 2)	Persistent non-weight-bearing lameness (score 5)	Atrophy of the musculature of the hip and thigh regions (score 2)	Osteoarthritis due to hip dysplasia
3.	Nine years old, 35 kg, female	Discomfort during extension of the hip (turns itself) (score 1)	Persistent severe weight-bearing lameness (score 4)	Decreased musculature of the hip and thigh regions (score 1)	Fracture of the femoral neck
4.	Two years old, 30 kg, female	Discomfort during extension of the hip (turns itself) (score 1)	Persistent severe weight-bearing lameness (score 4)	Decreased musculature of the hip and thigh regions (score 1)	Chronic coxofemoral luxation

A CT scanning examination of the pelvis and femur of each dog was performed using a 16 slice helical CT scanner (Siemens). Images were acquired as a volume with 0.5 mm voxels, 0.5 s rotation speed, 0.828 helical pitch, 512 × 512 matrix 120 kVp and 350 mA. The volume data were reconstructed with bone and soft tissue algorithms, as well as in isovolumetric transverse, sagittal, and frontal planes at 1–2 mm slice thickness. The CT images were available in a bone window (width: 2700 HU, level: 350 HU) and a soft tissue window (width: 400 HU, level: 40 HU). Volume bone algorithm data were imported into a three-dimensional (3D) workstation and 3D reconstructions of the hind limbs were created using a commercially available software. Using the 3D CT, images of the femoral head and neck were assessed. Revision surgery was performed for case 4 using deep gluteal muscle interposition.

Based on information from the owners combined with the results of clinical and CT evaluations, the outcome of the FHNOs was unsatisfactory in all four dogs. Functional limitations were present in all dogs despite appropriate aftercare and persisted without any trend to improvement. Severe weight-bearing lameness was observed in three dogs (score 4) and non-weight-bearing lameness in one dog (score 5). Atrophy of the musculature of the hip and thigh regions was observed in all dogs. Discomfort during extension of the hip occurred in all dogs, with severe pain expression in case 2.

**Fig. 1:**
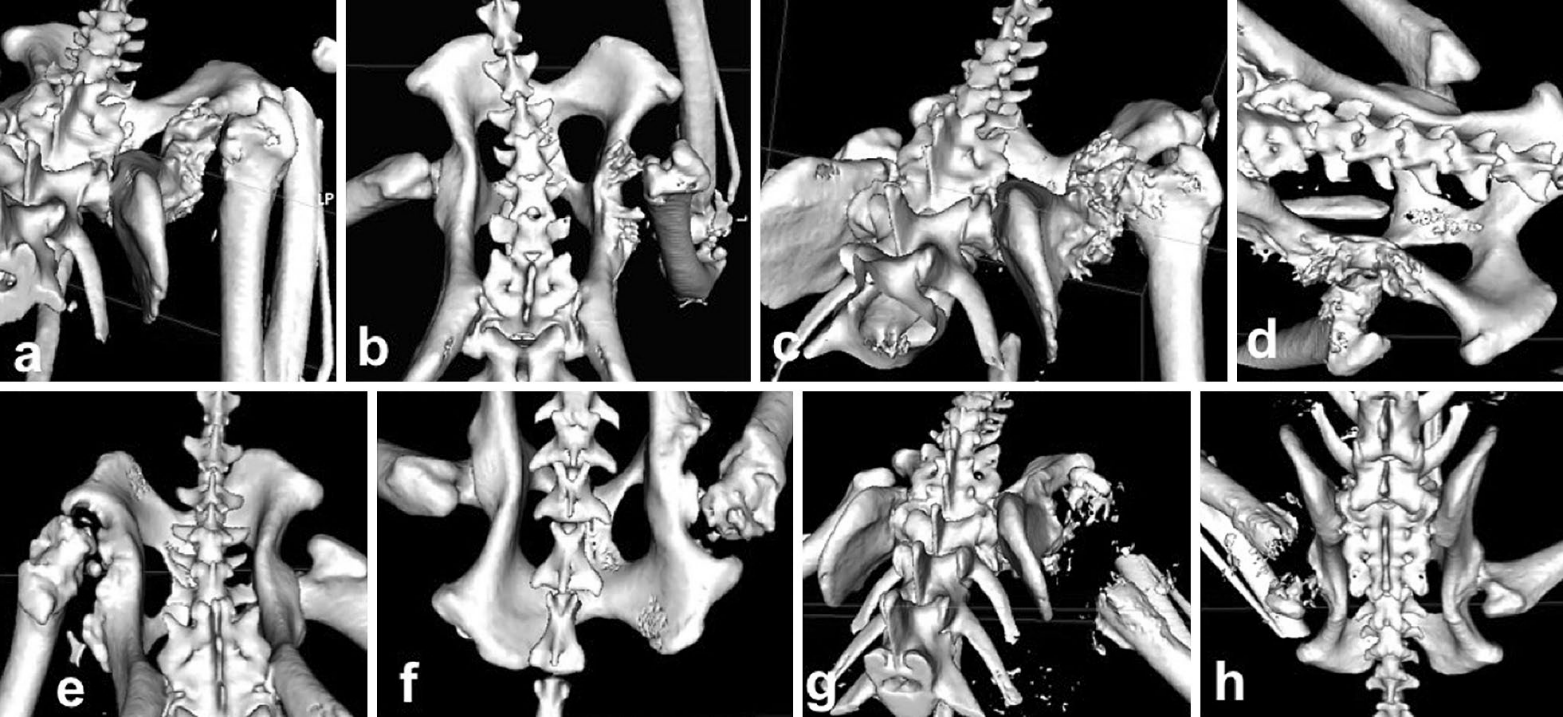
Three-dimensional reconstruction CT images of the hip joint post femoral head and neck ostectomy. Dorsal view: **a**, **c**, **e**, and **g**; Cranial view: **b**, **d**, **f** and **h**. **a**, **b** case 1. Note the insufficient bone removal, marked bony proliferation and unacceptable bone-on-bone contact. **c**, **d** case 2, Note the extensive remodelling, marked bony proliferation and unacceptable bone-on-bone contact. **e**, **f** case 3. Note the extensive remodelling, marked bony proliferation involving the femoral neck and acetabulum and free fragments. **g**, **h** case 4. Note the excessive ostectomy, bone lysis and free fragments

**Fig. 2:**
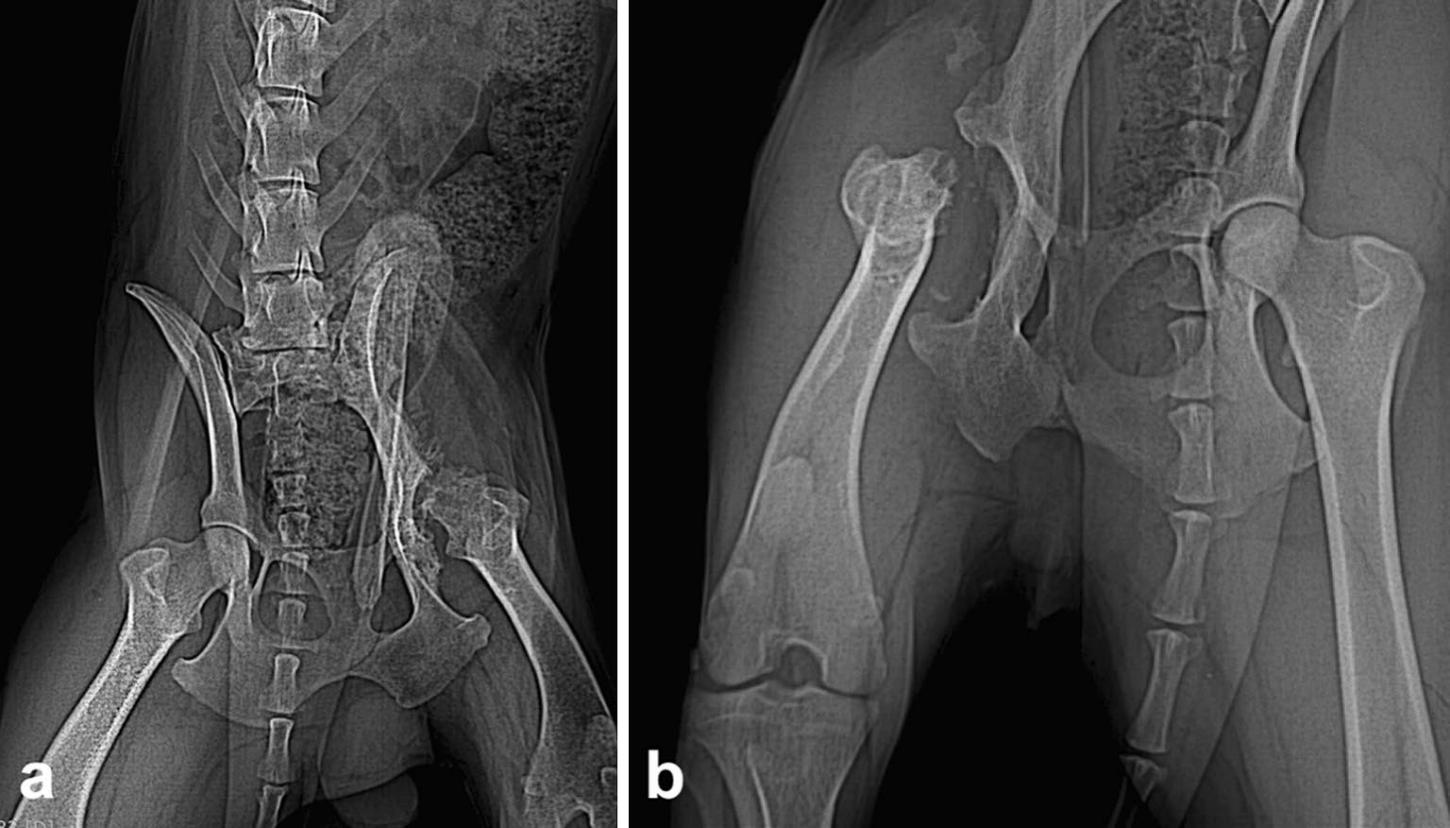
Ventrodorsal hip radiograph of case 1. This radiograph shows incomplete resection of the femoral neck and bony proliferation 10 months (**a**). Ventrodorsal hip radiograph of case 3. This radiograph shows extensive remodeling and bony proliferation involving the cut surface of the femoral neck and acetabulum after 11 months postoperative (**b**)

Extensive remodelling and marked bony proliferation involving the femoral neck and acetabulum was observed in three cases (Figs. 1a–f). Unacceptable bone-on-bone contact was observed in cases 1, 2 and 3, with insufficient bone removal (cases 1 and 3) and incomplete neck removal in case 2. In case 4, excessive ostectomy had probably been performed (Fig. 1g, h). Presence of free bone fragments was also observed in cases 3 and 4 (Fig. 1e–h). The surgical site had healed without any post-operative complications after deep gluteal muscle interposition in case 2. The cases were positioned incorrectly for radiology at different intervals after the surgeries (Fig. 2a, b). Three-dimensional CT reconstructions of the late bone remodelling offered major advantages in assessing inadequate bone-on-bone contact, comparing with conventional radiography. We consider that 3D CT reconstructions offer major benefits.

In all four dogs, FHNO failure was defined as an unsatisfactory limitation in function as noted by the owners, with lameness, pain, muscle atrophy, and limitation in range of motion observed on clinical examination. The ostectomies were performed by different veterinarians from private practice and we did not have access to details about the techniques applied. It could be argued that an unsatisfactory outcome was a consequence of technical errors. We consider that in three cases (cases 1, 2 and 3) there was CT evidence of inadequate bone removal. Based on CT findings in our patients, we agree with the idea that bone contact, which interrupts formation of a pseudarthrosis, is the main cause for poor outcome [7, 8, 16–20]. Residual femoral neck is frequently located at the poorly visualized caudal aspect and thus may be overlooked by an unexperienced surgeon [21].

It is probable that excessive ostectomy was performed on case 4. We cannot explain the preservation of the greater trochanter and absence of the bone between it and the rest of the femur, unless by a surgical error. The clinical significance of excessive bone removal seen in this case is unclear, but it has the potential of causing additional tissue trauma and altering the biomechanics of the post-operative pseudarthrosis [21]. Accidental removal of the lesser trochanter disrupts the attachment site of the iliopsoas muscle and is a cause of delayed recovery of the weight bearing function [22]. This could be another explanation for the poor functional outcome in case 4. However, some authors consider that complete excision of the femoral neck, including removal of the lesser trochanter, may improve results [23].

Clinically important complications necessitating surgical revision occurred, but only one owner (of case 2) accepted a sling musculature interposition as an alternative. Deep gluteal muscle interposition was performed, but no improvements were observed 3 months postoperatively. According to discussions with the owners 3 months postoperatively, the other three dogs presented the same functional disabilities. Total hip replacement alternative was rejected by all of the four owners due to cost-related considerations.

It was our perception that “bone-on-bone” contact between the degenerative femoral head and the degenerative acetabulum could be better assessed by CT scanning images. Conventional radiographic examinations are satisfactory for diagnostic, but cross-sectional imaging might sometimes be necessary for accurate characterization of periarticular osteophytes of the femoral neck as well as with regard to remodelling of the femoral heads and acetabulae, as the images provide additional information over plain radiographs.

We do not consider that the surgeons have to perform a CT scanning on every candidate for FHNO, but this additional information might be helpful for a revision surgery. The limitation of this study is the small number of dogs, thus we cannot consider CT scanning a crucial tool for clinical decision making.

It should be explained to all dog owners that surgical outcome is unpredictable when recommending FHNO, especially in large breed dogs.

## References

[1] Roush JK, Tobias KM, Johnston SA (2012). Surgical therapy of canine hip dysplasia. Veterinary surgery: small animal.

[2] Berzon JL, Howard PE, Covell SJ, Trotter EJ, Dueland R (1980). A retrospective study of the efficacy of femoral head and neck excisions in 94 dogs and cats. Vet Surg.

[3] Duff R, Campbell JR (1977). Long term results of excision arthroplasty of the canine hip. Vet Rec..

[4] Duff R, Campbell JR (1978). Radiographic appearance and clinical progress after excision arthroplasty of the canine hip. J Small Anim Pract.

[5] Duff R, Campbell JR (1978). Effects of experimental excision arthroplasty of the hip joint. Res Vet Surg..

[6] Gendreau C, Cawley AJ (1977). Excision of the femoral head and neck: the long-term results of 35 operations. J Am Anim Hosp Assoc.

[7] Lippincott CL (1984). Excision arthroplasty of the femoral head and neck utilizing a biceps femoris muscle sling. Part two: the caudal pass. J Am Anim Hosp Assoc.

[8] Off W, Matis U (1997). Ganganalyse beim Hund. Teil 2: Aufbau eines Ganglabors und bewegungsanalytische Untersuchungen. Tierärztl Prax..

[9] Lippincott CL (1981). Improvement of excision arthroplasty of the femoral head and neck utilizing a biceps femoris muscle sling. J Am Anim Hosp Assoc.

[10] Fitzpatrick N, Pratola L, Yeadon R, Nikolaou C, Hamilton M, Farrell M (2012). Total hip replacement after failed femoral head and neck excision in two dogs and two cats. Vet Surg.

[11] Tarvin G, Lippincott CL (1987). Excision arthroplasty for treatment of canine hip dysplasia using the biceps femoris muscle sling: an evaluation of 92 cases. Semin Vet Med Surg Small Anim.

[12] Jeffery ND (1993). Femoral head and neck excision complicated by ischiatic nerve entrapment in two dogs. Vet Comp Orthop Traumatol..

[13] Prostredny JM, Toombs JP, VanSickle DC (1991). Effect of two muscle sling techniques on early morbidity after femoral head and neck excision in dogs. Vet Surg.

[14] Lewis DD, Bellah JR, McGavin MD, Gaskin JM (1988). Postoperative examination of the biceps femoris muscle sling used in excision of the femoral head and neck in dogs. Vet Surg.

[15] Mann FA, Tangner CH, Wagner-Mann C, Read WK, Hulse DA, Puglisi TA, Hobson HP (1987). A comparison of standard femoral head and neck excision and femoral head and neck excision using a biceps femoris muscle flap in the dog. Vet Surg.

[16] Olmstead ML, Hohn RB, Turner TM (1983). A 5-year study of 221 total hip replacements in the dog. J Am Vet Med Assoc.

[17] Plante J, Dupuis J, Beauregard G, Bonneau NH, Breton L (1997). Long-term results of conservative treatment, excision arthroplasty and triple pelvic osteotomy for the treatment of hip dysplasia in the immature dog. Vet Comp Orthop Traumatol..

[18] Farrell M, Clements DN, Mellor D, Gemmill T, Clarke SP, Arnott JL (2007). Retrospective evaluation of the long-term outcome of non-surgical management of 74 dogs with clinical hip dysplasia. Vet Rec..

[19] Off W, Matis U (2010). Excision arthroplasty of the hip joint in dogs and cats—clinical, radiographic, and gait analysis findings from the Department of Surgery, Veterinary Faculty of the Ludwig-Maximilians-University of Munich, Germany. Vet Comp Orthop Traumatol..

[20] Montgomery RD, Milton JL, Horne RD, Coble RH, Williams JC (1987). A retrospective comparison of three techniques for femoral head and neck excision in dogs. Vet Surg.

[21] O’Donnell MD, Warnock JJ, Bobe G, Scholz RP, Wiest JE, Nemanic S (2015). Use of computed tomography to compare two femoral head and neck excision ostectomy techniques as performed by two novice veterinarians. Vet Comp Orthop Traumatol..

[22] Grisneaux JD, Dupuis J, Pibarot P, Bonneau NH, Charette B, Blais D (2003). Effects of postoperative administration of ketoprofen or carprofen on short- and long-term results of femoral head and neck excision in dogs. J Am Vet Med Assoc.

[23] Lippincott CL (1992). Femoral head and neck excision in the management of canine hip dysplasia. Vet Clin North Am..

